# Integrative miRNA-mRNA network analysis to identify crucial pathways of salinity adaptation in brain transcriptome of *Labeo rohita*

**DOI:** 10.3389/fgene.2023.1209843

**Published:** 2023-08-31

**Authors:** Nitin Shukla, Harshini Vemula, Ishan Raval, Sujit Kumar, Vivek Shrivastava, Aparna Chaudhari, Amrutlal K. Patel, Chaitanya G. Joshi

**Affiliations:** ^1^ Gujarat Biotechnology Research Centre, Gandhinagar, Gujarat, India; ^2^ Postgraduate Institute of Fisheries Education and Research, Kamdhenu University, Gandhinagar, Gujarat, India; ^3^ Central Institute of Fisheries Education, Mumbai, Maharashtra, India

**Keywords:** brain transcriptome, *Labeo rohita*, hub genes, miRNA-mRNA regulatory network, salinity adaptation

## Abstract

**Introduction:** Brain being the master regulator of the physiology of animal, the current study focuses on the gene expression pattern of the brain tissue with special emphasis on regulation of growth, developmental process of an organism and cellular adaptation of *Labeo rohita* against unfavourable environmental conditions.

**Methods:** RNA-seq study was performed on collected brain samples at 8ppt salt concentration and analyzed for differential gene expression, functional annotation and miRNA-mRNA regulatory network.

**Results:** We found that 2450 genes were having significant differential up and down regulation. The study identified 20 hub genes based on maximal clique centrality algorithm. These hub genes were mainly involved in various signaling pathways, energy metabolism and ion transportation. Further, 326 up and 1214 down regulated genes were found to be targeted by 7 differentially expressed miRNAs i.e., oni-miR-10712, oni-miR-10736, ssa-miR-221-3p, ssa-miR-130d-1-5p, ssa-miR-144-5p and oni-miR-10628. Gene ontology analysis of these differentially expressed genes led to the finding that these genes were involved in signal transduction i.e., calcium, FOXO, PI3K-AKT, TGF-β, Wnt and p53 signalling pathways. Differentially expressed genes were also involved in regulation of immune response, environmental adaptation i.e., neuroactive ligand-receptor interaction, ECM-receptor interaction, cell adhesion molecules and circadian entrainment, osmoregulation and energy metabolism, which are critical for salinity adaptation.

**Discussion:** The findings of whole transcriptomic study on brain deciphered the miRNA-mRNA interaction patterns and pathways associated with salinity adaptation of *L. rohita*.

## 1 Introduction

Evolution has provided fishes with an exceptional ability to adapt with the varying salinity conditions (Greenwell et al., 2003) which helps them survive in water with different salt concentrations. Fishes have been known to show detectable changes at cellular and molecular level due to change in the salt concentration in its surrounding environment. These changes in turn help them maintain homeostasis in hypersaline conditions (Tseng and Hwang, 2008). Researchers have found special osmosensory receptors in fish brain which helps them maintain osmolarity and provides sensitive feedback mechanism (Noble, 2008). In fish brain, osmoreceptors (neurons) are highly controlled by extracellular fluid osmolality (Bourque et al., 1994) and specifically hypothalamus and pituitary glands play key roles in osmotic homeostasis (Bernier et al., 2009). Anthropogenic activities and natural calamities have increased the salt percentage in the land recent time (Williams, 1999; Suzuki et al., 2002; Roache et al., 2006). This is causing the serious effects on aquatic ecosystem via ionic and osmotic stress on freshwater aquatic life. Rohu (*L. rohita*, Cyprinidae), is an important freshwater fish in India and other Asian countries (Mahapatra et al., 2016; Rasal et al., 2017). Rohu has significantly higher muscle protein content than other major carps Catla and Mrigal (Shakir et al., 2013). The species commands a good market price and consumer demand which makes it economically important.

Researchers in the past studied the effect of salinity concentration as environmental stressors on kidney and gills due to their pivotal role in osmoregulation. However, recent studies on regulatory and signalling mechanisms involved in osmoregulation strengthen the importance of brain function in modulation of the immune system, neurotransmitters, relevant receptors and regulatory proteins and environmental adaptation in relation to hypo or hyperosmotic stress (Kim et al., 2015; Liu et al., 2018; Wang et al., 2020).

MicroRNAs (miRNA) are small non-coding RNAs of approximately 22 nucleotides in length which are involved in post-translational regulation of mRNAs through RNA induced silencing complex (RISC) (Bartel, 2018). miRNA based gene regulation is omnipresent in animal kingdom (Berezikov, 2011). miRNAs are known to play important role in embryonic development, morphology, organogenesis (Wienholds et al., 2005; Kapsimali et al., 2007; Bizuayehu et al., 2012; Fu et al., 2013) and maintenance of osmotic regulation (Flynt et al., 2009; Yan et al., 2012a; 2012b).

Hence, the authors tried to understand the changes in the physiology of the animal brain by looking into the transcriptional changes in the brain during the hyper salinity treatment. The present study not only looked into the up and downregulated genes but also investigated hub genes and constructed integrated miRNA-mRNA regulatory networks to elucidate key molecular mechanisms underlying acclimatization of *L. rohita* under hyper salinity environment.

## 2 Materials and methods

### 2.1 Ethical statement

The animal study was reviewed and approved by Kamdhenu University, Gandhinagar, Gujarat. The guidelines of the CPCSEA (Committee for the Purpose of Control and Supervision of Experiments on Animals, Ministry of Environment and Forests (Animal Welfare Division) on care and use of animals and ARRIVE2.0 (Animal Research: Reporting of *In Vivo* Experiments) (du Sert et al., 2020) in scientific research were followed during the experiment.

### 2.2 Sample collection and salinity stress

Salinity stress experiment was conducted at Postgraduate Institute of Fisheries Education and Research, Kamdhenu University, Himmatnagar, Gujarat. Healthy rohu (*L. rohita*, Cyprinidae) fingerlings (>10 g) were acquired from a local freshwater farm and acclimatized to lab conditions in 150 L tanks (15 fingerlings/tank) for 7 days with continuous aeration at 27°C ± 5°C. Feeding was done three times a day at the rate of 5% body weight. Unused feed and faecal matter were siphoned out and 25% of water was replaced daily. Later the fingerlings were randomly divided into control and salinity treatment groups. The experiment was done in triplicate. Control group was maintained at 0ppt throughout the experiment, while for the treatment group the salinity was gradually increased (1ppt/day) to specified salinity (2, 4, 6 and 8ppt) by adding a solution (55ppt) of Red Sea Coral Pro Salt (Red Sea, United States). The salinity was checked with a salinity refractometer RES-10ATC (ATC, United States). The fingerlings were maintained at a particular salinity for 6 days and on the 6th day they were transferred to next level salinity concentration. The process was repeated until salinity concentration reached to 8ppt on the 32nd day from the start of the experiment. Then fishes were sampled randomly and brain tissues were dissected out aseptically. Samples were stored at −80°C in RNAlater until further use. RNA isolation was done in duplicates for control (B1C8 and B2C8) and treatment (B1T8 and B2T8) samples.

### 2.3 RNA extraction and construction of library

RNeasy Plus Mini Kit was used for the extraction of total RNA. The RNA isolation was done as per the manufacturer’s protocol (Qiagen, Germany). The quality of RNA (OD260/280) was checked in the QIAxpert™ instrument (Qiagen, Germany) and RIN value was determined by the Agilent 2100 Bioanalyzer™ system (Agilent technologies, California, United States).

Low Input RiboMinus™ Eukaryote System v2 (Thermo Fisher, Massachusetts, United States) was used to carry out rRNA depletion and TruSeq™ Total RNA Library Prep Kit (Illumina, California, United States) was used for library preparation. RNA-seq paired end sequencing library was sequenced in Novaseq 6000 (2 bp × 59 bp read length).

### 2.4 Quality control and mapping of raw data

The raw binary base call (BCL) files were converted into FASTQ files using Illumina Dragen (--bcl-conversion-only) and the sequencing files were evaluated using FASTQC (http://www.bioinformatics.babraham.ac.uk/projects/fastqc/). The raw sequences data have been submitted to NCBI Short Read Archive (SRA) (Table 1). The high-quality reads (i.e., Q-value >30), were used to map against reference genome of *L. rohita* (Jayanti breed) (GenBank assembly accession: GCA_004120215.1) downloaded from NCBI with Dragmap (https://github.com/Illumina/DRAGMAP). FeatureCounts was used to count the abundance of individual reads that mapped to genomic features.

**TABLE 1:** Details of Biosample and Short Read Archive (SRA) submission of *L. rohita* brain transcriptome to NCBI along with mapping statistics of transcriptome sequences.

Parameters	Fresh water (control)		Saline water (treatment)	
	B1C8	B2C8	B1T8	B2T8
Bioproject	PRJNA853878			
Biosample accession No.	SAMN29932459	SAMN29932461	SAMN29932460	SAMN29932462
SRA accession No.	SRR20601656	SRR20601654	SRR20601655	SRR20601653
Q30 bases	1,158,807,543 (94.03%)	1,996,914,814 (96.33%)	1,329,488,568 (95.33%)	1,541,464,277 (95.09%)
Input Reads	20,976,004	35,271,622	23,732,914	27,584,838
Mapped reads	18,925,878 (90.23%)	32,583,614 (92.38%)	21,371,278 (90.05%)	25,618,759 (92.87%)
Unmapped reads	2,050,126 (9.77%)	2,688,008 (7.62%)	2,361,636 (9.95%)	1,966,079 (7.13%)
Average read length	58.75	58.77	58.76	58.77

### 2.5 Differential expression analysis, gene ontology and KEGG pathway enrichment

Each transcript obtained after mapping was subjected for identification of differential gene expression in treatment group with comparison to control group using fragments per kilobase of exon per million mapped reads (FPKM). Differentially expressed genes (DEGs/DE mRNAs) between salinity treated and control groups were analysed with EdgeR (Empirical analysis of Digital Gene Expression) package from Bioconductor https://bioconductor.org/packages/3.15/bioc/html/edgeR.html, 12). *p*-value < 0.05 and log fold change (LogFC) > 0.5 or < −0.5 were used as threshold parameters to identify significantly expressed DEGs (logFC represents log2-fold-change). For enrichment and ontology of significant DEGs, GO and KEGG (Kyoto encyclopedia of genes and genomes database) was performed using GOATOOLS [https://github.com/tanghaibao/goatools, (Klopfenstein et al., 2018)] and KOBAS-i (v-3.0) (Bu et al., 2021) with FDR correction method (Benjamini and Hochberg) and statistical method (hypergeometric test/Fisher’s exact test).

### 2.6 PPI network analysis for identification of hub genes

Protein-protein interaction among differentially expressed genes was studied using STRING network analysis (https://string-db.org) with medium confidence interaction score of 0.4. Further, Cytoscape (3.9.1) was used to visualize the PPI network. CytoHubba (https://apps.cytoscape.org/apps/cytohubba), plugin of Cytoscape was used to identify the hub genes based on maximal clique centrality (MCC) algorithm. MCC was reported to be most effective algorithm in identification of hub genes with increased sensitivity and specificity (Chin et al., 2014). In our study, genes with top 20 MCC scores were considered as hub genes.

### 2.7 Putative mature miRNA identification and differential expression of miRNA

Paired end fastq files were merged and converted to fasta format. Further, collapsed reads were generated from the merged fasta files. Collapsing identical reads is beneficial for miRNA identification because miRNA length (16–24 bp) is lesser then sequence length (150 bp) (Baras et al., 2015). Identified putative mature miRNAs in *L. rohita* according to method followed by Mastakani et al. (2018) with some modifications as discussed below. The sequences of the mature miRNAs were obtained from the miRBase database (https://www.mirbase.org) for all teleostei species. Mature miRNA sequences were used as query sequences for offline BLASTn against the generated collapsed reads with parameters, i.e., E-value cut off 1E-1, percentage of identity ≥95. BLASTn output files were filtered based on miRNA sequences having minimum of 16 nt and maximum of 24 nt in length.

For differential expression of miRNA analysis, read count matrix was computed from collapsed read files and analysed with EdgeR package from Bioconductor. *p*-value < 0.05 and log fold change (LogFC) > 0.5 or < −0.5 were used as threshold parameters to identify significant differentially expressed miRNAs.

### 2.8 miRNA target gene prediction and construction of miRNA-mRNA regulatory network

Potential targets for differentially expressed miRNAs were identified using miRanda (3.3a) tool, which works on local homology between mature miRNA-sequence and query target gene sequence. In this study, we used coding sequences of *L. rohita* as target sequence obtained from NCBI (GenBank assembly accession no: GCA_004120215.1_ASM412021v1). Differentially expressed mRNAs targeted by differentially expressed miRNAs were considered as miRNA-mRNA (gene) regulatory pairs for further analysis and miRNA-mRNA regulatory network was constructed by Cytoscape (3.9.1).

## 3 Results

### 3.1 Transcriptome characteristics and mapping statistics

**FIGURE 1:**
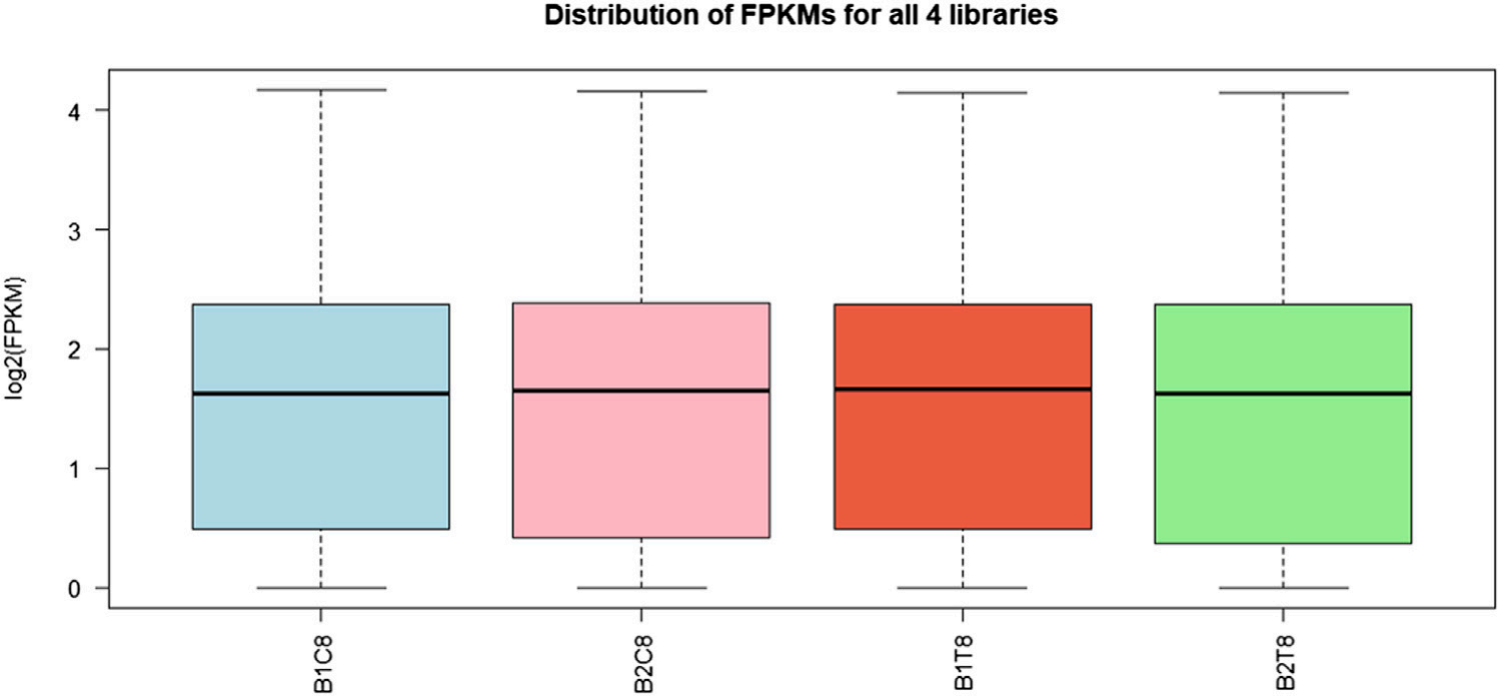
Box plot for read count distribution in normalized data. The solid horizontal line represents the median and each box contains lower and upper quartiles.

Post sequencing approximately 22 gigabytes paired end data was generated from control and salinity treated samples. A total of 107 million raw reads were obtained, which included 56 million reads for freshwater control samples and 51 million reads for salinity treated samples. Alignment with reference genome resulted in the mapping percentage ranging from 90.05% to 92.87% in control and salinity challenged groups. Summary statistics of transcriptome sequences are presented in Table 1. Normalized reads were generated using trimmed mean of M-values (TMM) method from read counts as represented in Figure 1 and the same were used for differential gene expression analysis.

### 3.2 Differential gene expression

**FIGURE 2:**
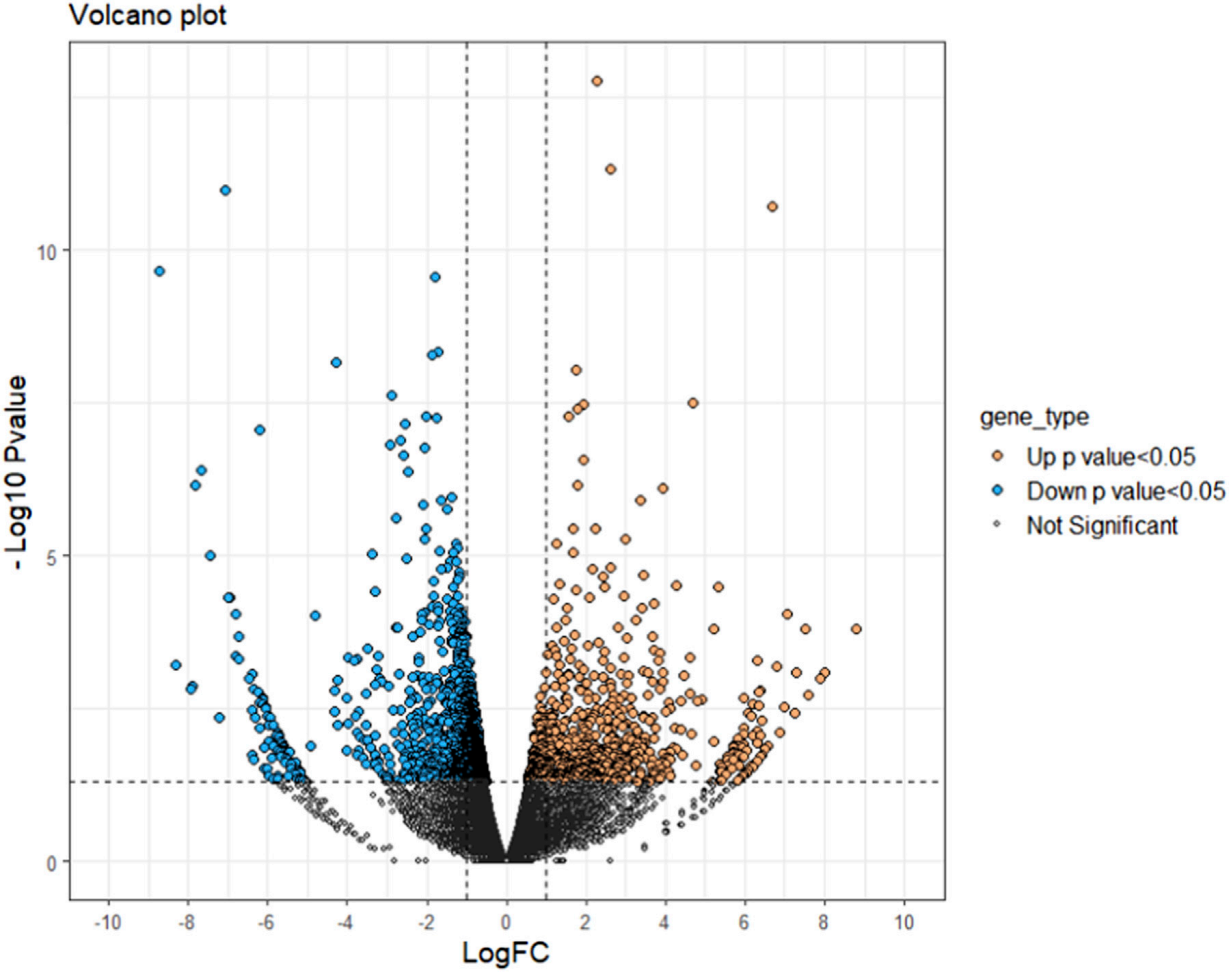
Volcano plot of differentially expressed genes identified between control and salinity treated rohu fish. The X-axis represents log fold change value and Y-axis indicates −Log10 *p*-value. The black colour dots indicate no significant difference, blue and orange dots indicate significantly down and upregulated genes, respectively.

A total of 37,462 transcripts were obtained in both control and treatment groups. With the threshold of *p*-value < 0.05 and LogFC > ±0.5, a total of 2,450 transcripts showed significant differential expression in saline treated samples in comparison with control. A complete list of significant down (1,716) and up (734) regulated genes were provided in the Supplementary Table S1. The volcano plot representing the difference in fold change among statistically significant genes is given in Figure 2.

### 3.3 Identification and pathway enrichment of hub genes

**FIGURE 3:**
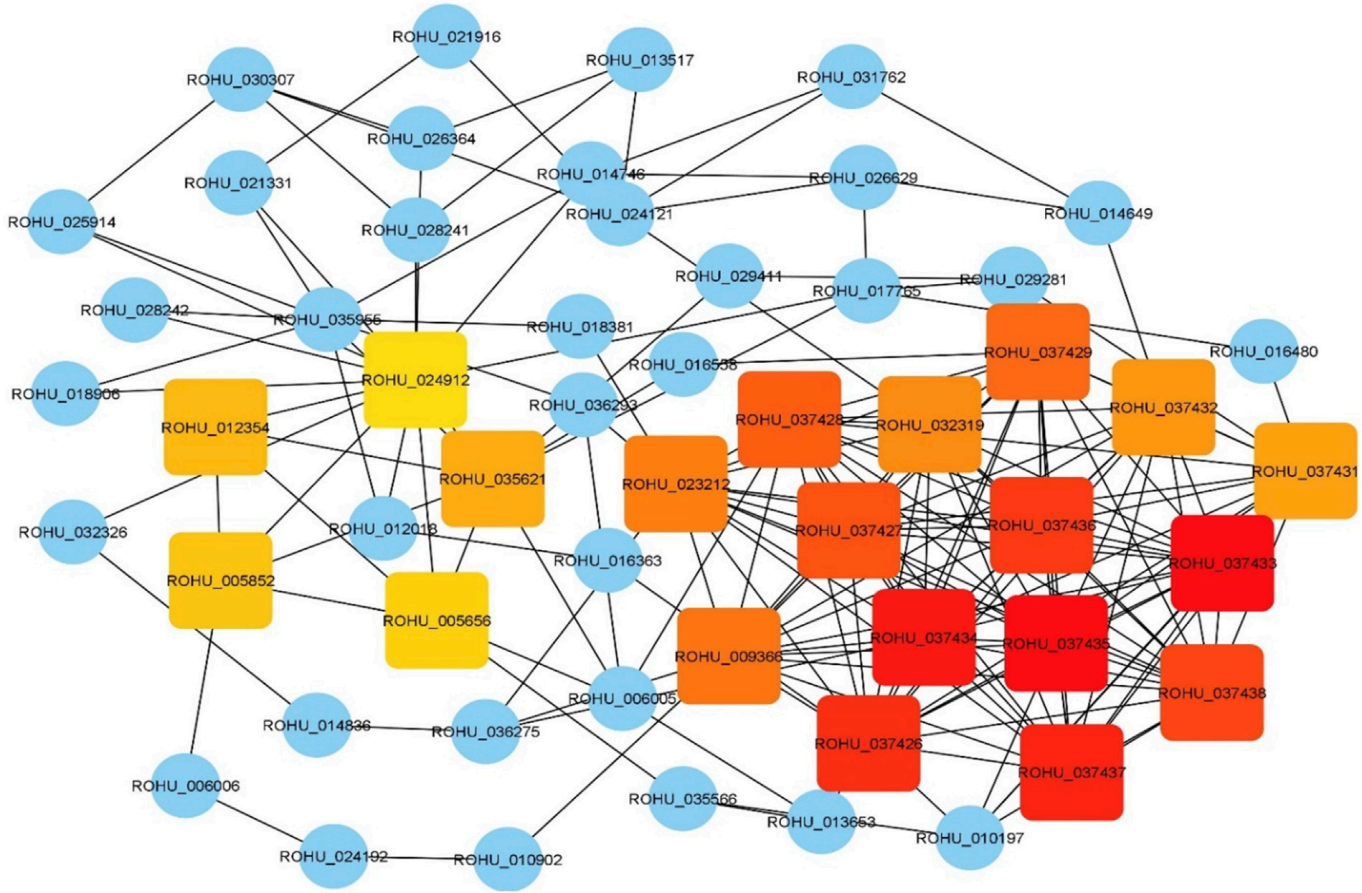
Hub genes identified using CytoHubba plugin with maximal clique centrality (MCC) algorithm on Cytoscape. Edges represent protein-protein interaction. Red nodes represent genes with highest MCC score and yellow nodes represent genes with low MCC scores. Blue nodes represent the genes that are directly interacted with hub genes.

Top 20 hub genes identified from PPI network are presented in the Table 2 (Figure 3). Pathway enrichment analysis revealed that hub genes are enriched in 11 KEGG pathways, namely, oxidative phosphorylation, metabolic pathways, focal adhesion, regulation of actin cytoskeleton, cardiac muscle contraction, MAPK signalling pathway, ErbB signalling pathway, insulin signalling pathway, gap junctions, VEGF signalling pathway and calcium signalling pathway.

**TABLE 2:** Top 20 hub genes with highest MCC score in PPI network of brain transcriptome data along with gene names and log fold change values.

Gene id	Gene name	Log fold change
ROHU-037433	NADH dehydrogenase subunit 3	1.94
ROHU-037435	NADH dehydrogenase subunit 4	1.78
ROHU-037434	NADH dehydrogenase subunit 4L	2.26
ROHU-037437	NADH dehydrogenase subunit 6	0.81
ROHU-037426	NADH dehydrogenase subunit 1	1.20
ROHU-037436	NADH dehydrogenase subunit 5	1.25
ROHU-037438	Cytochrome b	1.76
ROHU-037427	NADH dehydrogenase subunit 2	1.66
ROHU-037428	Cytochrome c oxidase subunit I	0.54
ROHU-037429	Cytochrome c oxidase subunit II	1.53
ROHU-009366	NADH dehydrogenase [ubiquinone] iron-sulfur mitochondrial	2.67
ROHU-023212	NADH dehydrogenase [ubiquinone] 1 alpha sub complex subunit 5	0.70
ROHU-032319	NADH dehydrogenase [ubiquinone] 1 alpha sub complex subunit 1	0.70
ROHU-037432	Cytochrome c oxidase subunit III	1.80
ROHU-037431	ATP synthase F0 subunit 6	1.67
ROHU-035621	SHC-transforming 1 isoform X1	−0.70
ROHU-012354	Adenylate terminal-differentiation specific-like protein	1.27
ROHU-005852	Platelet-derived growth factor receptor beta-like isoform X1	−0.60
ROHU-005656	Fibroblast growth factor 1	−5.67
ROHU-024912	SHC-transforming 2-like isoform X1	−1.78

### 3.4 Differential expression of miRNAs and their target DE mRNAs

BLASTn against mature miRNA sequences of teleostei revealed several potential miRNA hits against control and salinity treated samples (Supplementary Table S2). Differential expression of miRNA analysis showed that a total of six miRNAs were having significant *p*-value < 0.05, namely, oni-miR-10712, oni-miR-10736, ssa-miR-221-3p, ssa-miR-130d-1-5p, ssa-miR-144-5p and oni-miR-10628. All these miRNAs were found to be downregulated (Table 3). A total of 1540 DE mRNAs, including 326 upregulated and 1,214 downregulated genes, were identified as final set of miRNA-mRNA regulatory pairs (Supplementary Table S3). Data for individual miRNA is presented in Table 3.

**TABLE 3:** Differentially expressed miRNAs in brain transcriptome of *L. rohita* with their log fold change values and target DE mRNAs.

miRNA	Log fold change	*p*-value	Target DE mRNA		
			Up	Down	Total
oni-miR-10712	−4.52	1.85E−06	45	239	284
oni-miR-10736	−1.01	0.002	68	294	362
ssa-miR-221-3p	−4.63	0.004	25	62	87
ssa-miR-130d-1-5p	−1.93	0.029	108	263	371
ssa-miR-144-5p	−4.60	0.031	51	137	188
oni-miR-10628	−2.10	0.042	29	219	248

### 3.5 miRNA-mRNA regulatory network

**FIGURE 4:**
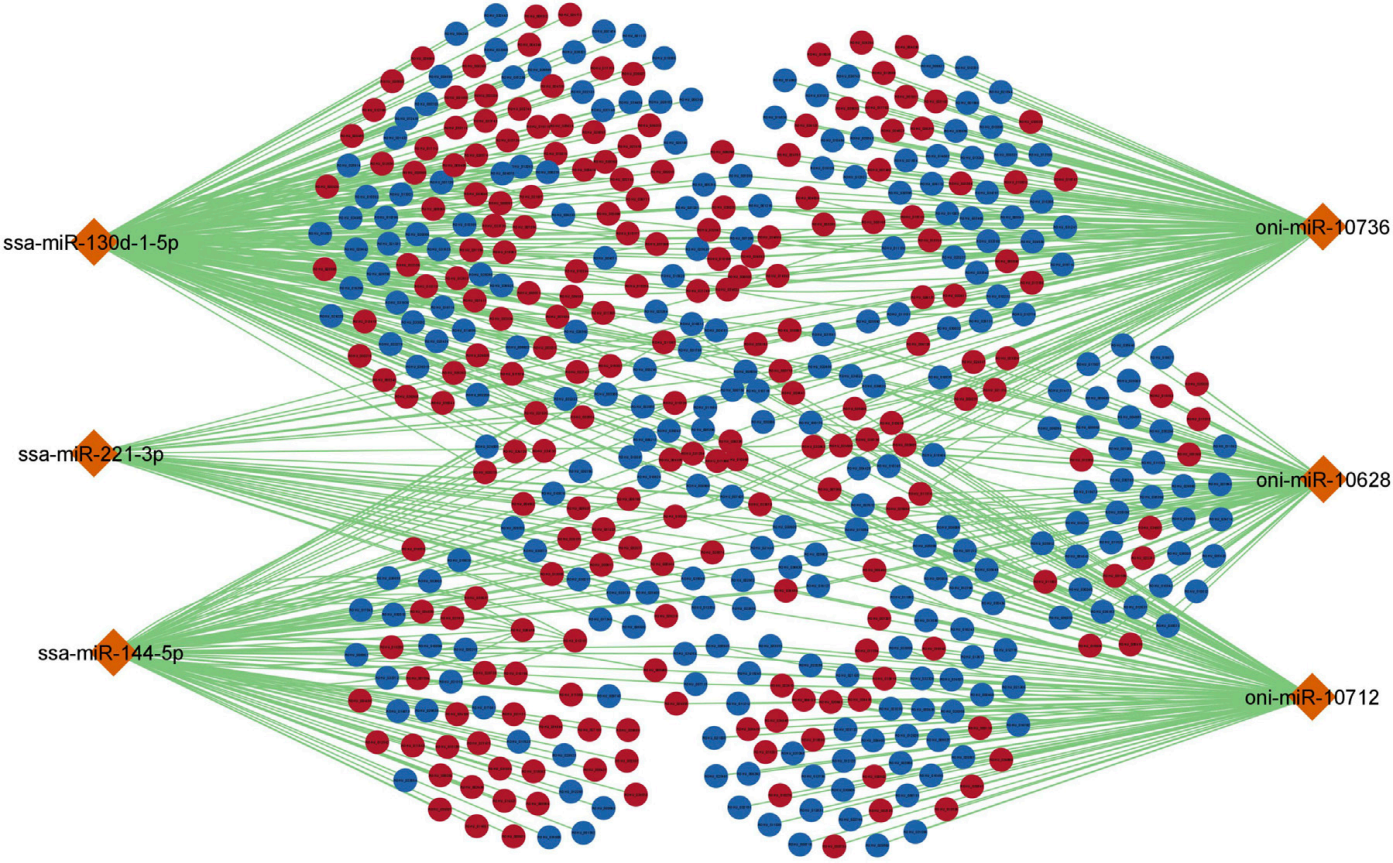
miRNA-mRNA regulatory network for differentially expressed miRNAs and mRNAs. The orange colour diamond represents DE miRNAs, dark red and dark blue elliptical shapes represents up and down regulated mRNAs respectively. The generated network has 477 nodes and 810 edges. Each node represents individual miRNA/mRNA.

To further understand the regulatory relationship between differentially expressed miRNAs and mRNAs, miRNA-mRNA regulatory network of 477 nodes and 810 edges was constructed. Several common up and downregulated mRNAs were targeted by DE miRNAs as shown in Figure 4. ssa-miR-130d-1-5p and oni-miR-10736 showed the highest connectivity with 371 and 362 DE mRNAs respectively (Table 3).

### 3.6 Gene ontology and KEGG pathway enrichment analysis of DE mRNAs

**FIGURE 5:**
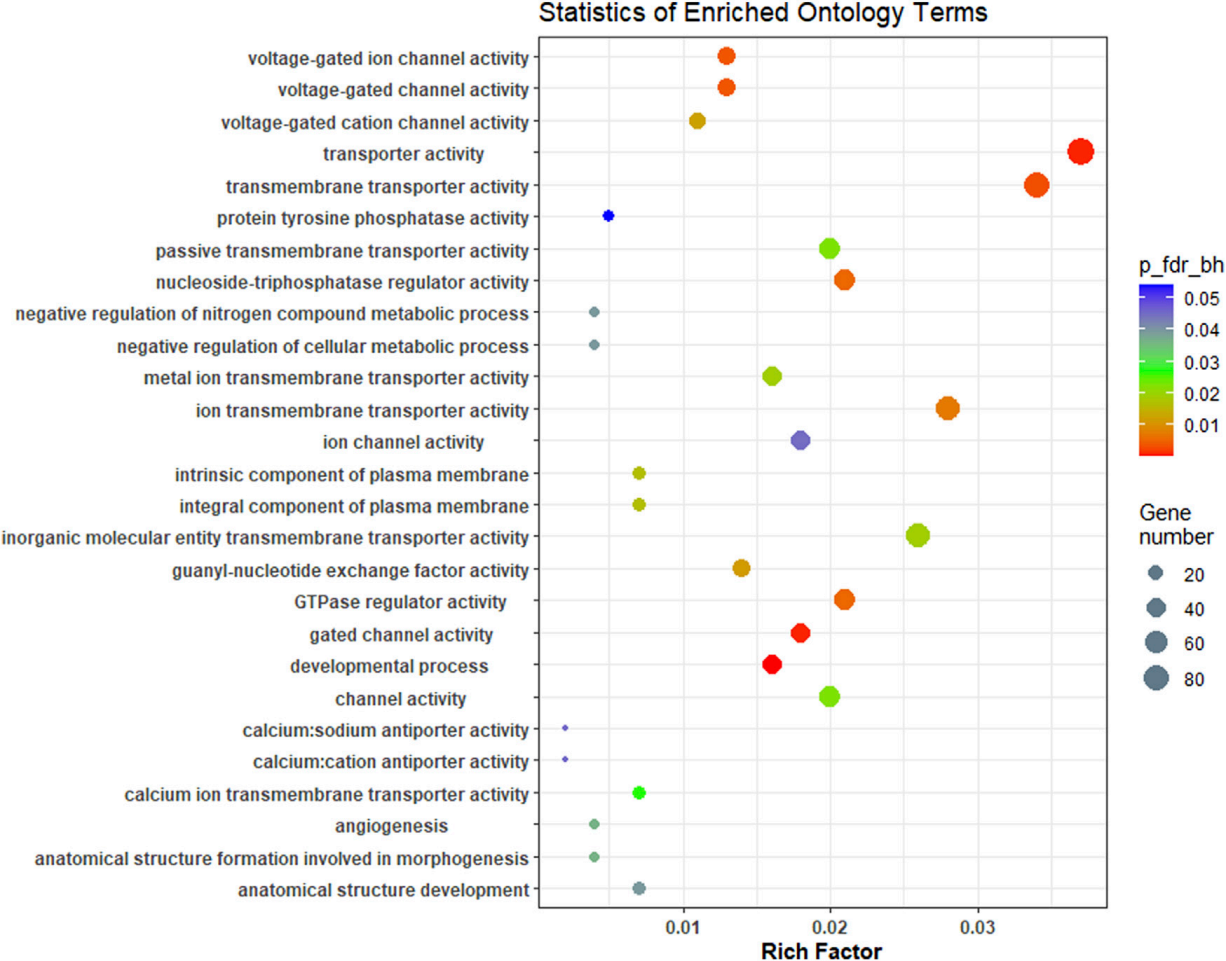
Overview of Top GO terms enriched (biological process, cellular component and molecular function) in brain transcriptome of *L. rohita* under hypersalinity stress. The Y-axis signifies ontology and the X-axis indicates the enrichment ratio. The colour shows FDR (Bejamini Hochberg) and size of the dots represents the number of significant genes involved in each corresponding process.

**FIGURE 6:**
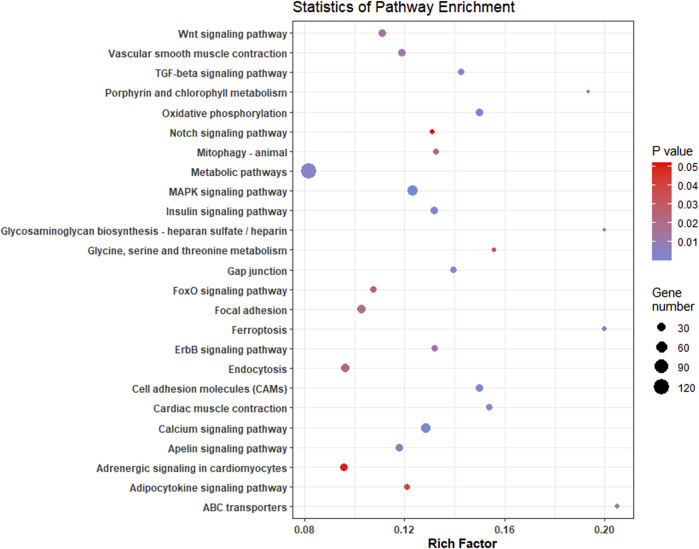
Scatterplot of enriched KEGG pathways of significant differentially expressed genes in *L. rohita* under salinity effect. Rich factor is the ratio of the number of DEGs to the total number of genes in a given KEGG pathway. The colour of the dots represents the range of *p*-value and size represents the number of genes involved in a pathway.

A complete list of all enriched gene ontology terms with GO ids and genes associated are given in Supplementary Table S4. Most of the significantly enriched GO terms were involved in transporter activity and signal transduction (Figure 5). KEGG pathway analysis of complete list of DEGs showed enrichment of 25 pathways (Figure 6; Table 4; Supplementary Table S5). The KEGG pathway enrichment of DE mRNAs targeted by DE miRNAs is given in Supplementary Table S6. All the genes enriched in MAPK, Wnt, p53, calcium, FOXO and TGF-beta signalling pathways were regulated by oni-miR-10736 and oni-miR-10628 miRNAs, whereas genes involved in environmental information processing pathways were regulated by all mentioned miRNAs in Table 3 except for ssa-miR-221-3p miRNA. We also observed that ssa-miR-130d-1-5p miRNA commonly targeted the key genes of energy metabolism such as hexokinase-1, 6-phosphofructo-2-kinase fructose-2,6-bisphosphatase 4-like isoform X1 (PFKEB1), aldehyde dehydrogenase and fatty acid synthase. Polyketide synthase (PKS) enzyme involved in synthesis of poly unsaturated fatty acids was found to be regulated by oni-miR-10736 and ssa-miR-221-3p. Among DE miRNAs oni-miR-10712 was found to mediate the expression of several crucial genes involved in various forms of programmed cell death such as apoptosis, necroptosis and ferroptosis.

**TABLE 4:** Significantly enriched KEGG pathways obtained using significantly expressed DEGs of *L. rohita* brain transcriptome in 8ppt salinity treated group vs. *L. rohita* of freshwater control.

KEGG pathway	Number of DEGs associated	Total No. of background genes in pathway	*p*-value
MAPK signaling pathway	46	373	5.05E−05
Calcium signaling pathway	37	288	0.000126317
Oxidative phosphorylation	21	140	0.000603421
Cell adhesion molecules (CAMs)	21	140	0.000603421
Cardiac muscle contraction	18	117	0.001110306
Insulin signaling pathway	22	167	0.002056876
Gap junction	18	129	0.002904985
TGF-beta signaling pathway	17	119	0.003016153
Metabolic pathways	123	1507	0.003999203
Ferroptosis	9	45	0.004307335
ABC transporters	8	39	0.006104485
Apelin signaling pathway	21	178	0.007956198
Vascular smooth muscle contraction	19	160	0.010555284
ErbB signaling pathway	14	106	0.01209365
Wnt signaling pathway	21	189	0.014124852
Focal adhesion	25	243	0.017918842
Glycosaminoglycan biosynthesis-heparan sulfate/heparin	6	30	0.018469869
Porphyrin and chlorophyll metabolism	6	31	0.020965737
Endocytosis	30	311	0.02156453
Mitophagy - animal	11	83	0.023868175
FOXO signaling pathway	18	167	0.027852695
Glycine, serine and threonine metabolism	7	45	0.033575978
Adipocytokine signaling pathway	11	91	0.040268471
Adrenergic signaling in cardiomyocytes	21	219	0.050403705
Notch signaling pathway	8	61	0.051914554

## 4 Discussion

Several studies have been carried out to assess the salinity tolerance of rohu (*L. rohita*, Cyprinidae) using different salinities and treatment conditions (Pillai et al., 2003; Islam et al., 2014; Hoque et al., 2020). Islam et al. (2014) reported that the rohu fingerlings were acclimatized to various salinities up to 12ppt and collected samples at each salinity up to 90 days at 15 days intervals, where mortality was seen only at 8ppt and above. They reported 85% relative percent survival (RPS) at 8ppt on the 30th day whereas complete mortality at 10ppt on the 4th day. Fulton’s condition factor was also significantly affected at 8ppt. Clearly, rohu fingerlings experience physiological stress at this salinity, and hence this value was selected here in our study to identify salinity stress response genes of rohu. The acclimation and sampling procedure was designed to collect samples well before mortality sets in (6 days at 8ppt), but enough for the salinity response genes to be expressed. Brain tissue was selected for this study as it is the centre of the nervous system which controls the physiological and behavioural mechanisms of the individual (Liu et al., 2018). The biochemical changes persuaded by high salt concentration in the brain revealed modifications in several regulatory mechanisms as an adaptive response of *L. rohita* to salinity. An extensive analysis of gene ontology and KEGG terms provided the insights into several pathways which are significantly enriched in the brain transcriptome under stress.

### 4.1 Altered signal transductions in *L. rohita* under hypersalinity effect

The enrichment of neuronal signalling pathways, viz., calcium, FOXO, PI3K-AKT, p53, TGF-beta and Wnt signalling pathways was observed in *L. rohita* maintained under high salt concentration. Calcium ion is a ubiquitous secondary messenger that has critical importance in transmission of depolarizing signals (Brini et al., 2014), release of neurotransmitters, synaptic transmission (Kawamoto et al., 2012), energy production (Fink et al., 2017) and cell survival (Kawamoto et al., 2012). In *L. rohita*, the differential expression of calcium voltage-dependent channels, sodium-calcium exchanger isoforms, calcium calmodulin kinases, muscarinic acetylcholine receptors, vascular endothelial growth factors, P2X purinoceptors and solute carrier family genes of calcium transport involved in calcium signalling pathway might be related to maintenance of neuronal plasticity and ATP production in response to environmental stress. Downregulation of FOXO3 transcription factor, insulin like growth factor receptor (IGF1R), insulin receptor and 33 genes involved in PI3K-AKT pathway was observed. The insulin/insulin-like growth factor phosphatidylinositol-3-kinase-AKT/FOXO pathway hastens the cellular senescence in cultured human dermal fibroblasts (Kim et al., 2005). Under salinity stress, cellular senescence might be an alarming signal to prevent further multiplication of the abnormal cells and protect the individual (Kumari and Jat, 2021). Downregulated PI3K-AKT/mTOR pathway also shows neuroprotective effect by promoting autophagy (Wang et al., 2014). Under salinity stress, autophagy might improve neuronal cell survival and protein homeostasis by lysosomal mediated degradation of misfolded proteins and removal of damaged organelles (Naves et al., 2012).

In p53 signalling pathway, downregulation of BCL2 causes apoptosis through intrinsic mitochondrial mediated pathway and MDM2 by enhancing the p53 activity (Shi and Gu, 2012) in response to programmed cell death of abnormal cells under salinity effect. Transforming growth factor beta (TGF-β) proteins have a recognizable role in neuroprotection followed by various brain tissue injuries (Dobolyi et al., 2012). TGF-β2 works interactively with fibroblast growth factors to regulate homeostasis of motor neurons (Jiang et al., 2000), while TGF-β1 can instruct the neurons from growth state to synaptogenic state (Dobolyi et al., 2012). TGF-β signalling pathway is also an important component in the antioxidative mechanism in brain cells under oxidative stress (Chen et al., 2021). Hence, the enriched TGF-β signalling pathway inferred the neuroprotective function in *L. rohita* under salinity environmental stress.

The Wnt/β-catenin signalling pathway is a complex network in association with several cellular mechanisms for sensing change in the environment and regulated accordingly (Christodoulides et al., 2015). Under salinity effect, β-catenin interaction with FOXO transcription factors exhibits protective function against oxidative stress (Essers et al., 2005), maintenance of tissue architecture and cell polarity by forming interlinking between cadherins and actin cytoskeleton (Ozawa et al., 1989; Schambony et al., 2004). p53 provides an interlink between Wnt-signalling to cellular stress pathways (Christodoulides et al., 2015) and mutual interaction between Wnt/β-catenin signal transduction with hypoxia inducible factor-1 α (HIF-1α) in regulation of stress response under hypoxia due to high salt concentration (Xia et al., 2019).

### 4.2 Immune response to salinity effect

Antigen processing and presentation and phagosome pathway are two immune pathways enriched in *L. rohita* brain. Upregulation of cathepsin S, K and B in the phagosome pathway reflected the lysosomal protein degradation. It is followed by enrichment of the MHC class I antigen presentation pathway, as it is one of the distinct pathways for the presentation of antigenic peptides to CD8^+^ and CD4^+^ T cells (Jensen, 2007). The upregulation of MHC class I related proteins, H-2 class II histocompatibility antigens, antigen peptide transporter-2 and tumor necrosis factor was observed. The increased expression might be related to acquired immunity of *L. rohita* to salinity stress. The same observations were also reported in *Oreochromis niloticus* under long term hypersalinity stress (Liu et al., 2018). In this study, genes were upregulated related to necroptosis pathway, namely, TNFSF10, caspase 1, cytochrome B, CAMK2B, and SLC25A4 (Table 2). In fish, necroptosis is also responsible for stimulating physiological functions such as the immune defence, inflammatory reactions and plays an important role in the process of maintaining cellular homeostasis (Xia et al., 2021). Hence, upregulation of pathways might be related to immune response of the *L. rohita* to salinity stress.

### 4.3 Environmental information processing under salinity stress

In our study, four significantly changed pathways are associated with processing of the information in response to environmental stress, viz., neuroactive ligand-receptor interaction pathway, ECM-receptor interaction, cell adhesion molecules and circadian entrainment. Neuroactive ligand-receptor interaction pathway comprehends all the ligands and receptors of the cell membrane for signal transduction (Lauss et al., 2007) and were differentially expressed under salinity stress in *L. rohita*. The similar results were also observed in Nile tilapia (Liu et al., 2018) under high salinity experimental conditions. Cells communicate through cell to cell contact or via extracellular matrix (ECM) (Edeleva and Shcherbata, 2013). The external cellular stressors transduce the signals from signalling molecules through cell signalling receptors and cell adhesion molecules (CAMs) present on the cell surface interconnected with ECM and communicate the information (Edeleva and Shcherbata, 2013). Neurotransmitters and their receptors are important in sensing salinity stress and signalling modulations in fish physiology (Liu et al., 2018). Glutamate is the major excitatory neurotransmitter of the central nervous system and in our study differential expression of ionotropic and metabotropic glutamate receptors were observed. Glutamate plays a key role in rapid inter neuronal communications (Mustafayev and Mekhtiev, 2013). Circadian entrainment, maintains the organism’s homeostasis under external or internal stressors through physiological fight or flight system and enables the organism to cope up with the additional stress stimuli (Thompson and Spencer, 1966; Johnson et al., 1992; Grissom and Bhatnagar, 2009). In our study, enrichment of the circadian entrainment pathway reflected the adaptive mechanism of *L. rohita* under high salt concentration.

### 4.4 Response regulation in *L. rohita* under hypersalinity stress

Osmoregulatory genes such as solute carrier (SLC) family genes, claudins, gap junction related genes, sodium-chloride dependent creatine transporter-1 (SLC6A8) and sodium-chloride dependent GABA transporter-2 (SLC6A13) were differentially expressed in our study (Supplementary Table S1). SLC genes are membrane bound transporter proteins, play key role in osmoregulation, whereas SLC6A8 and SLC6A13 are involved in transport of neurotransmitters (Christie, 2007; Bergeron et al., 2008). Downregulation of enzymes involved in glycolysis was observed, specifically pyruvate dehydrogenase enzyme, which helps in conversion of pyruvate to Acetyl-CoA. However, Acetyl-CoA is the precursor for the citric acid cycle for ATP generation. This led to generation of Acetyl-CoA through an alternative pathway of fatty acid beta oxidation through enzyme Acyl-CoA synthetase (Zhang et al., 2019). Apart from this there was also over-expression of enzyme polyketide synthase, key limiting enzyme in production of polyunsaturated fatty acids through PKS pathway (Metz et al., 2001) and several isoforms of apolipoproteins. This inferred that lipid metabolism plays an important role in energy production to maintain osmoregulation under salinity stress in *L. rohita*. The free amino acids can be metabolized and be a source of ATP and involved in regulation of intracellular osmotic pressure (Venkatachari, 1974; Tseng and Hwang, 2008; Nguyen et al., 2016). In the present investigation, upregulation of cationic amino acid transporter-2 (SLC7A2), responsible for cellular uptake of arginine, lysine and ornithine (Saroglia and Liu, 2012) was identified.

Enrichment of ferroptosis (downregulation of transferrin receptor-1), an iron dependent non-apoptotic cell death due to accumulation of lipid reactive oxygen species (Dixon et al., 2012) under high salt concentration was observed in *L. rohita*. However, glutathione peroxidase 4A (GPX4), which enables detoxification of lipid hydroperoxides by interacting with lipids in membranes, over-expression reflects the protective function (Ursini and Bindoli, 1987) under salinity effect. Transformation of *L. rohita* from freshwater to saltwater might cause disposal of xenobiotics due to environmental shifts (Nguyen et al., 2016). Candidate gene, Glutathione-S-transferase (GST), involved in detoxification of xenobiotics by quenching reactive molecules and catalyzing glutathione conjugation to hydrophobic and electrophilic substrates, protects the cells against the oxidative burst (Kumar and Trivedi, 2018). The same reports were also made in transcriptomic study of *Anguilla anguilla* (Kalujnaia et al., 2007) and *Pangasianodon hypophthalmus* (Nguyen et al., 2016).

## 5 Conclusion

In summary the present study provides insights into differentially expressed genes and enriched pathways in brain transcriptome of *L. rohita* under hyper salinity stress. The present study has found correlation between the miRNA dysregulation and differential expression of various transcripts. The study has identified key miRNAs which are master regulators of DEGs involved in metabolic processes like osmoregulation and energy production which are one of the most important factors for salinity adaptation, whereas oni-miR-10736 was found to regulate the genes enriched especially in protein processing in endoplasmic reticulum under salinity stress. miRNAs were also shown to regulate the differentially expressed mRNAs enriched in pathways related to various signal transductions, environmental information processing, immune response, osmoregulation, energy metabolism and detoxification, which are critical for salinity adaptation in *L. rohita*. In the present study, miRNA identified through total RNA sequencing and miRNA database specific to *L. rohita* is not available. Hence, novel miRNAs specific to *L. rohita* may be missing.

## Data Availability

The original contributions presented in the study are included in the article/Supplementary Material, further inquiries can be directed to the corresponding authors.
